# Selection for Tameness in Red Junglefowl Recapitulates Genetic Loci Associated With Domestication‐Related Brain Composition

**DOI:** 10.1111/mec.17788

**Published:** 2025-05-19

**Authors:** Carlos Guerrero‐Bosagna, Fábio Pértille, Zahra Moradinour, Rebecca Katajama, Maria Luisa Martin Cerezo, Rie Henriksen, Per Jensen, Dominic Wright

**Affiliations:** ^1^ Physiology and Environmental Toxicology Program, Department of Organismal Biology, Evolutionary Biology Center Uppsala University Uppsala Sweden; ^2^ IFM Biology Linköping University Linköping Sweden

**Keywords:** adaptation, behaviour/social evolution, ecological genetics, life history evolution, natural selection and contemporary evolution, quantitative genetics

## Abstract

Domestication involves huge phenotypic shifts via strong directional selection. The resulting changes, often termed the Domestication Syndrome, typically encompass numerous traits; however, the most universal of these are changes in reduced fear of humans (tameness) and brain composition. To assess how early domestication selection may have focused on tameness and its interaction with brain composition, a Red Junglefowl ( *Gallus gallus* ) population (the wild progenitor of the domestic chicken) was used to create two lines bidirectionally selected for fear of humans over eight generations of selection. These selection lines were then used to make an intercross population. Using a combination of genome‐wide mapping in the intercross and between‐line analysis of the selection lines, we show that the genetic loci for tameness co‐localise with genetic loci for brain composition and anxiety behaviour. Furthermore, the detected loci for brain composition also co‐localise with brain composition loci identified in a separate wild × domestic intercross. These results indicate that tameness and brain composition are either pleiotropic or genetically linked, and that tameness selection appears to recapitulate the same loci that have been selected by domestication itself. Therefore, selection for increased tameness could be the initial selection pressure driving the core of the domestication syndrome.

## 1 Introduction

Animal domestication can be viewed as a large‐scale, natural biological experiment in which humans are responsible for the selection of traits that are being propagated in a population (Price 2002) and, as such, it is a special case of evolution driven by humans. This was realised already by Darwin, who used it as proof‐of‐concept when developing the theory of evolution by natural selection (Darwin 1859). Domestication has been defined as the process whereby animals change genetically and phenotypically in response to the selection pressure associated with a life under human supervision (Jensen and Wright 2022). This includes both directed human selection, but, of course, also natural selection that is likely to have been important, not least during the early phases of domestication (Jensen and Wright 2022). Individuals that were not able to cope with the captive environment close to humans are not likely to have thrived, regardless of human intervention. Consequently, early domestication in animals is thought to have been initially driven largely by selection on tameness, where tameable individuals were more likely to accommodate to life among humans (Belyaev 1979; Agnvall et al. 2017; Bélteky et al. 2016).

A striking consequence of domestication is the evolution of a set of phenotypic traits that are very similar across species, commonly referred to as the domestication syndrome (Wright 2015; Wright et al. 2020a, 2020b). This syndrome includes, for example, changes in body size, loss of pigmentation, and increased reproductive capacity (Price 2002). It has been suggested that most of these traits have evolved as correlated side effects to the above‐mentioned selection for increased tameness and may thus have appeared early in the domestication history of any species. Lately, experimental selection studies have been conducted to test this hypothesis, and studies of, e.g., silver foxes (Trut et al. 2009), rats (Albert et al. 2009), mink (Malmkvist and Hansen 2001) and chickens (Agnvall et al. 2012) have revealed that some, but not all, correlated selection responses indeed are consistent with the evolution of the domestication syndrome. For example, selected only for reduced fear of humans, silver foxes started to resemble domesticated dogs with, among other traits, floppy ears, white spots, and characteristic whimpering for human attention after only a few generations (Trut et al. 2009). However, it also remains equivocal how strong this evidence is (Lord et al. 2020a) and how widespread the domestication syndrome is (Wright et al. 2020a; Lord et al. 2020a).

One of the defining aspects of the domestication syndrome is a reduced brain size relative to body size compared to wild conspecifics (Wright 2015; Wright et al. 2020a). According to the mosaic brain hypothesis, changes in brain size can happen through selection on individual regions of the brain as a response to selection on specific behaviours (Barton and Harvey 2000). We have previously compared brain size between Red Junglefowl, *Gallus gallus* , (the progenitor of modern domestic chickens) and domesticated White Leghorn layers, *Gallus gallus domesticus*, and found that the domesticated breed has larger absolute brain mass, but a smaller brain size relative to body mass (Henriksen et al. 2016). However, the use of allometric scaling (always presenting brain size in respect to body size) is now not always considered strictly correct (Willemet 2013; Schoenemann 2006; Montgomery et al. 2010), and this can bias results. For example, in the case of a wild × domestic intercross that was used to map the genetic loci giving rise to the inter‐population variation in total and relative brain size in chickens, as well as body weight, the genetic architectures (i.e., the location of the QTL affecting inter‐population variation) were entirely separate between brain size (both total and relative) and body weight (Henriksen et al. 2016). In such cases, the use of relative brain size can actually conflate the results. In this case, the loci for larger brain size actually came from the White Leghorn (domestic) parent, but the effects were not seen in the parental birds, as the huge increase in body size in domestics masked these effects. Only when an intercross was used (and hence the body weight and brain size QTL were permuted into different combinations) could this be parsed.

Not only is brain size altered during domestication, but brain composition also undergoes radical shifts, not least in the chicken (Racicot et al. 2024). The sizes of different parts of the brain vary greatly, largely independently of each other. For example, the cerebellum is proportionally larger in domestic chickens (around a 15%–20% increase), as are the cerebral hemispheres, whilst the optic lobes are proportionally smaller (Henriksen et al. 2016). These differences in size reflect actual variation in the number of neuronal cells present, i.e., these altered brain regions are not simply packed with non‐neuronal cells, but represent actual changes in neuronal density (Cunha et al. 2022).

Through differential selection on tameness (fear of humans), we have previously found that adult relative brain size was reduced after only a few generations of selection in Red Junglefowl, in some ways mirroring the effects found in domesticated chickens (Agnvall et al. 2017). In particular, the cerebellum was larger in birds selected for low fear. These results could support the hypothesis that tameness may be the principal driver for the evolution of the brain compositional and behavioural aspects of the domestication syndrome. However, whether this is through pleiotropic effects or due to linked loci remains unknown and requires investigation.

Given that domestication involves rapid responses to selection, it has been posited that DNA methylation and other epigenetic mechanisms could play a role in facilitating the rapid phenotypic alterations that occur during domestication (Höglund et al. 2020a, 2024; Pértille et al. 2019). For example, in the chicken, though domestication occurred over 8000 years ago (Fumihito et al. 1994, 1996), the most rapid phenotypic changes occurred within the last 50 years, when the breeds were split into broilers (meat production) and layers (egg production). By using a wild × domestic advanced intercross, Höglund et al. demonstrated that a small number of key regions appear to regulate thousands of methylated DNA regions throughout the genome, with methylation also appearing to be an important mechanistic regulator of gene expression, with over a third of eQTL detected in this intercross showing a correlation with methylation (Höglund et al. 2020a). With the apparent importance of DNA methylation in regulating gene expression in the domestic chicken, and the massive trans regulatory hotspots, it could well be that such methylation changes also occur during the rapid and intense artificial selection of tameness. A previous analysis of the selected lines used here that looked at hypothalamus tissue found 33 significant differentially expressed genes between the lines and 22 1 kb methylation windows that were differentially methylated (Bélteky et al. 2016, 2018; Belteky et al. 2017). Additionally, we have previously shown that sperm DNA methylation appears to influence the occurrence of SNPs, with the emergence of SNPs in CpGs increasing with phylogenetic distance (Pértille et al. 2019).

Despite the central role of speciation in biology, the molecular mechanisms affected by species divergence are largely unknown (Burton 2022). To investigate this, and how the response to selection can affect multiple traits, we used an artificial selection model in chickens that mimics a proposed initial target of domestication selection, namely fear of humans or tameness. In order to explore the possible genetic underpinnings of correlated selection responses to tameness, and thereby to shed light on the mechanisms by which reduced fear of humans affects the domesticated phenotype, we generated a three‐generation intercross line between two selection lines of ancestral Red Junglefowl, bred during eight generations for high versus low fear of humans as assessed in a standardised behavioural test (Katajamaa and Jensen 2021). In this intercross, we assessed the scores for fear of humans as well as size and composition of the brain, which allowed us to conduct a broad quantitative trait locus (QTL) analysis to locate genomic regions affecting these traits. Since we have previously conducted similar studies comparing ancestral Red Junglefowl with modern domesticates, we can analyse possible overlaps of genomic regions affecting similar traits, thus further advancing the insights into the possible role of tameness as a driver of the domestication syndrome. In addition to this, genetic divergence between the selected lines used to generate the intercross was also assessed to assist in identifying differentially fixed regions arising due to selection. As epigenetic mechanisms are increasingly thought to have an active role in evolutionary processes (Guerrero‐Bosagna 2024; Jensen and Wright 2024) we also assessed whether selection on phenotypes would also concordantly select germline epigenotypes. DNA methylation was therefore also assessed between the selection lines, enabling us to identify differentially fixed methylation and the patterns of methylation that occur in the different lines, as well as which methylated genes are present in the QTL regions in the intercross population.

## 2 Materials and Methods

### 2.1 Ethical Note

All experimental protocols were approved by the Linköping Regional Committee for Ethical Approval of Animal Research, licences no. 50‐13, 14916‐2018, 10492‐2023. The experiments were conducted in accordance with the ARRIVE guidelines and relevant regulations.

### 2.2 Animals and Housing

The animals used were based on a selection experiment, with Red Junglefowl divergently selected for high and low fear of humans over eight generations, with the fifth selection generation selected to investigate fixed genetic and epigenetic differences between the lines. For the selection line comparison, these populations are referred to as Selection‐line 5th generation High (S5H), Selection‐line 5th generation Low (S5L), and Selection‐line 5th generation Intermediate (S5I). The selection lines were continued for a further three generations, before being used to create an intercross, initially *F*_1_, with the *F*_2_ and *F*_3_ used for a Quantitative Trait Loci analysis (QTL analysis). See below for a description of the breeding protocols of the selection lines as well as the intercross. All birds were bred and kept using the same housing routines. After hatch, each bird was weighed and wing tagged, then housed in groups in floor pens with feed and water ad lib, wood shavings, and heat lamps for the first three weeks. When five weeks old, the birds were moved to the breeding facility and kept in groups of 40–50 in three‐level aviary pens (3 × 3 × 3 m) with perches and nest boxes as well as access to an outdoor aviary (3 × 3 × 3 m) and *ad libitum* access to feed and water. A 12:12 h dark:light cycle was maintained in both housing facilities.

### 2.3 Breeding of Selection Lines

We used two different zoo populations of Red Junglefowl as the origin of our selection lines, with these being derived from a line taken from the Copenhagen zoo and the other from the Götala research station (see Håkansson et al. 2007 for more information on the background of these populations). These were first interbred for two generations to create an outbred parental generation from which we separately bred the least and most fearful individuals. Fearfulness towards humans, fear‐of‐human (FOH) score, was determined individually in all animals using a standardised behaviour test (described in more detail below). More information about the breeding programme for the selection lines, including population sizes, can be found in Agnvall et al. (2012).

### 2.4 Breeding of the Intercross Line

For details regarding breeding of the intercross line, see Katajamaa and Jensen (2021). Briefly, four individuals (two males and two females with reciprocal design) from each selection line, in total eight individuals, from the eighth selected generation were used to create an intercross line, generation *F*_1_. In the *F*_1_ and *F*_2_ generations, birds were randomly bred to generate an *F*_3_ generation intercross. In the *F*_1_ generation, six females and six males were randomly selected to create breeding pairs, and in the *F*_2_ generation, 19 females and 19 males were randomly assigned into breeding pairs. In the *F*_3_ generation, 134 individuals were hatched. All birds in the different generations were tested to obtain FOH scores. The advantage of breeding past the *F*_2_ generation is that the number of recombination events increases, and hence shortens the length of the haplotype blocks, which increases the precision in finding genetic correlations between quantitative traits such as behaviours (Darvasi and Soller 1995). The recombination rate in chickens is, on average, 1% and the average haplotype block is 350 kb, which gives a rough estimate of the strength of associations that can be expected in each generation (Ka‐Shu Wong et al. 2004).

## 3 Intercross Analysis (Behavioural and Morphological Tests, Markers, Analysis)

### 3.1 Phenotypic Analyses

#### 3.1.1 Fear of Human (FOH) Test

A total of 159 birds (70 females, 89 males) were tested in a standardised behavioural test to determine fearfulness towards humans and obtain a FOH score when the birds were 12 weeks old. This was done in the same way for all birds in all generations and was identical to the test used in breeding the selection lines. In the *F*_3_ generation, we tested 47 females and 66 males, whilst in the *F*_2_ generation, 24 females and 30 males were tested. The test has been described in detail previously (Agnvall et al. 2012). Each bird was tested individually in an arena (100 × 300 × 210 cm), in which the behaviour in response to an approaching human was recorded. At the start of the test, the bird was placed on its own in the middle of the arena, while the arena was kept in darkness. The observer, who at the same time was the human stimulus, entered and remained still for 60 s. When the lights were turned on, observations began. Every ten seconds during a total of 180 s, the fear level of the chicken was recorded according to a 5‐point scale, where score 1 was the least fearful and 5 the most fearful reaction. After 60 s, the observer moved to the middle zone and after 120 s, the observer moved to the last zone, adopting the same upright and motionless posture. After 180 s, the observer attempted to touch the chicken without chasing it and noted the fear reaction to that in addition to the fear score. The overall FOH score for each bird was calculated as the mean of all the sampling points of the test and could therefore range on a continuous scale from 1 to 5.

#### 3.1.2 Flash/Startle Test

A total of 102 chicks (45 female, 57 male) were tested in a fear habituation and memory test, consisting of two test trials, conducted 24–28 h apart. With each of the two trials, a single chick was placed in a closed, evenly illuminated test arena, measuring 25 × 25 × 30 cm. In the floor, we placed an LED light source emitting blue light, and after 30 s in the arena, the chicks were exposed to a series of five brief (1 s) light flashes with a 30 s interval in between. Thirty seconds after the last flash, the chick was removed and replaced by the next animal.

The tests were video recorded, with the startle responses of the chicks scored using the Observer software (Noldus Inc). A five‐point scale was used to score the response, with the lowest score (Price 2002) being where no reaction to the flash was observed, and the highest (Agnvall et al. 2017) being the maximal fear reaction. To validate the scoring, we selected a random sub‐sample (10%) of the videos, which were then scored by two independent observers. The observer score correlation was *r*_s_ = 0.91 (*p* < 0.001). Details and an ethogram for this methodology can be found in Malmkvist and Hansen (2001).

An overall measure of the intensity and dissipation of the fear response was calculated by using the area under the curve (AUC) for each chick on each of the test days. The AUC was calculated by adding the areas under the curve for each of the five 30 s intervals. A lower AUC therefore means lower overall reaction scores.

#### 3.1.3 Open Field Test

A total of 102 chicks (45 female, 57 male) were tested in a standard open field (OF) test when they were 4 weeks old. The arena was a totally enclosed, soundproof square test arena with even lighting, measuring 80 × 120 cm. The test arena was virtually divided into two sections, a centre zone measuring 40 × 80 cm and periphery consisting of the area outside of the centre zone. Each chick was placed individually in a corner of the arena with the lights turned off. When the lights were turned on, the birds were allowed to freely explore the arena for 5 min after which the test was finished. EthoVision (Noldus, version XT 10) was used for automatic recording of the movements in the arena. Total distance moved and time spent in the centre were recorded. The test was performed twice on two consecutive days (24–28 h in‐between) and the averages from the two test sessions were used for the statistical analysis.

#### 3.1.4 Brain Composition

At 32 weeks of age, all birds were weighed, then culled by rapid decapitation. Brains were removed and dissected into four parts according to the protocol used by Henriksen et al. (2016): cerebral hemispheres, optic lobes, cerebellum, and the remaining part containing, e.g., brainstem and hypothalamus (referred to as the midbrain). Each brain region was immediately weighed to within 0.001 g (Sagitta, model 210 g/1 mg). Previous research has found a high correlation between wet mass and volume (Jensen and Wright 2022).

#### 3.1.5 Body Weight

The chickens were weighed when culled using a scale with 0.01 g precision.

### 3.2 SNP Marker Generation for Intercross Birds

Parental animals used to generate the intercross, *F*_2_, and *F*_3_ individuals were all genotyped using a RADseq methodology. Adapters were designed following the quaddRAD protocol outlined in Franchini et al. (2017). Restriction enzyme overhangs were modified for SbfI and MseI. 8 bp‐long barcodes were designed using EDITTAG (Faircloth and Glenn 2012), with a minimum Levenshtein distance of 4 nucleotides, GC content of 40%–60% and avoiding sequences that were self‐complementary and contained more than two adjacent, identical bases. From the tags suggested by EDITTAG, sequences that reconstructed SbfI and MseI restriction sites were removed manually. 15 tags were selected for the inner adapters and 10 for the outer adapters.

Inner adapters were prepared by annealing each single‐stranded oligonucleotide with its complementary strand. 5 μL of each bottom and top strands at 100 μM were mixed with 40 μL of annealing buffer (50 mM NaCl, 10 mM Tris‐Cl, pH 8.0), heated to 97.5°C for 2.5 min and cooled at a rate of 1°C per minute down to 21°C. Once prepared, the adapters were kept at −20°C and used within 2 weeks. 10 ng of genomic DNA was then digested and ligated to the inner adapters in a single‐step 40 μL reaction containing 4 μL 10× CutSmart buffer, 1.5 μL Mse1 (10 U/μL), 0.75 μL Sbf1 (20 U/μL), 4 μL ATP (10 mM), 1 μL T4 DNA ligase (400 U/μL), 0.75 μL of each quaddRAD_i5n and quaddRAD_i7n inner adapters (10 μM), ddH2O to 17.25 μL and incubated for three hours at 30°C in a thermocycler. The reaction was stopped with 10 μL of 50 mM EDTA. Samples were purified and double size‐selected using 0.5× and 0.8× Sera‐Mag SpeedBeads solution (GElifesciences, Marlborough, MA, USA) containing 10 mM Tris base, 1 mM EDTA, 2.5 M NaCl, 20% PEG 8000 and 0.05% Tween 20 (pH 8.0), washed twice with 80% ethanol, and eluted in 30 μL of ddH2Ol. To introduce the outer barcoded adapters, an indexing PCR was carried out in a 50 μL reaction containing 4 μL of each i5 and i7 primers (5 mM), 1 μL of dNTPs (10 mM), 0.5 μL of purified water, 10 μL enhancer, 10 μL of 5× Q5‐HF Buffer, 0.5 μL of Q5‐HF DNA Polymerase (New England Biolabs, Frankfurt am Main, Germany) and 20 μL of template DNA. After an initial denaturation step of 30 s at 98°C, the PCR reaction was carried out in 15 cycles (15 s at 98°C, 30 s at 67°C and 645 s at 72°C) and a final elongation at 72°C for 5 min. Purification was performed using 0.8× Sera‐Mag SpeedBeads solution (GElife‐ sciences, Marlborough, MA, USA), washed twice with 80% ethanol, and DNA was eluted in 30 μL ddH2O. Samples were multiplexed by combining 10 ng of each sample to form the final library for each plate. Libraries were then size‐selected to 300–600 bp prior to sequencing, and sequenced on an Illumina Novaseq PE150, 15G raw data per sample (Illumina Inc., San Diego, CA, USA).

### 3.3 Statistical Analysis

SNPs generated using the ddRADseq genotyping protocol were analysed and called using the STACKS v2.68 program (Catchen et al. 2013, 2011). STACKS was used to process the RADtags and call the SNP genotypes. The refmap flag was used to annotate the genotypes to Galgal6 and then export the SNP genotypes. A total of 10,621 SNPs were generated for analysis.

### 3.4 Genome Wide Association Analysis (GWAS)

GWAS was performed using the program GWASpoly v2.13 (Rosyara et al. 2016, The Plant Genome). This program controls for population structure using a random polygenic effect (the K model—Yu et al. 2006). To reduce “proximal contamination”, whereby a marker would be included both in the random effect, GWASpoly uses a leave‐one‐chromosome‐out method (Yang et al. 2014), in which the covariance relatedness matrix is based on all chromosomes bar the one the marker being tested is present on. To reduce multiple testing, marker curation was used to exclude markers below a certain threshold. Rather than use minor allele frequency, GWASpoly uses a maximum genotype frequency instead, which is more appropriate for heterozygous panels in particular. In this case, we used a minimum of five individuals required to carry the minor allele (with a value of 1–5/177 used in the parameter file for GWASpoly). Both an additive and a 1‐dominance model were used to test for marker significance. A False Discover Rate method was used to generate the significance threshold, with a 5% false discovery rate (FDR rate) used for significance, and a 10% FDR rate used as a suggestive threshold. The function get.QTL was used to select all suggestive and significant QTL, with a window threshold set at 5e6 bp (i.e., the most significant SNP present in a 5e6 bp window is returned for each QTL). The function fit.QTL takes all significant QTL that were detected and fits them into a single linear model to calculate the individual *p*‐value of each of the QTL in the combined model. This function has the limitation that you cannot select a subsection of QTL to be dropped, but still gives an idea of how significant each SNP is when placed within a linear model of all significant SNPs, with these *p*‐values being present in Table S1. Confidence intervals are often problematic to estimate with a GWAS analysis, and although we report the highest SNP in the above window threshold, given that we are using an *F*_2_/*F*_3_ population we should have fairly large confidence intervals. Therefore, when ascertaining whether QTL overlap, we use ±2 Mb window from the significant SNP location. This interval was chosen as the chicken has a rather high recombination rate when compared to humans, with one cM equal to 350 Kb. Therefore, 2 Mb is approximately 5.7 cM, so this interval is 11.4 cM. This is a rather narrow interval for an *F*_2_/*F*_3_ intercross; however, it does make the probability of an overlap between QTL more conservative. Conversely, care must be taken when solely using these confidence intervals to identify candidate genes.

### 3.5 Brain–Behaviour Correlations

Correlations between brain composition and behaviour and between brain composition were performed using the linear model function in R, with sex added as a covariate where significant.

### 3.6 Genetic and Epigenetic Differences Between the Selected Populations

Genetic and epigenetic differences between the selection lines used to generate the intercross population were assessed to identify genomic regions that were partly or fully fixed between the selection lines, and also DNA methylation changes that were also differentially fixed between the lines. We used the fifth generations of the selection lines (low, intermediate and high), with these generations continued for a further three generations of selection prior to producing the intercross. In the case of the epigenetic differences, these were measured in sperm to identify heritable epigenetic changes. To assess methylation changes, we used a Genotyping By Sequencing Methylation DNA immunoprecipitation (GBS‐MEDIP) protocol, see below. In summary, in our approach, we performed different contrasts in order to identify differences among the individuals from the 5th generation of different selection lines (S5H and S5L) and between them and the parentals (P0). We used samples from parental (*n* = 20), 5th generation high selection birds (*n* = 7), 5th generation intermediate birds (*n* = 4) and 5th generation low selection birds (*n* = 11) for these analyses.

## 4 Selection Lines Analysis (SNP Analysis, DNA Methylation Analysis)

### 4.1 DNA Extraction and GBS‐MeDIP Procedure

Sperm ejaculates were obtained from male chickens via cloacal massage and then frozen at −20°C until further processing. The detailed procedure for the processing of sperm samples has been previously described (Pértille et al. 2019). Briefly, for the purification of sperm heads from somatic contamination, sperm cells were incubated with collagenase, sonicated for 5 s at 60% amplitude, and then subjected to three series of vortexing (30 s) and centrifugation (3 min; 4000 *g*; RT) (Pértille et al. 2019). DNA was extracted from the purified sperm cells as previously described (Pértille et al. 2019) via Proteinase K digestion followed by protein precipitation, and DNA precipitation with isopropanol. The extracted DNA was then used for constructing sequencing libraries via the GBS‐MeDIP protocol (Rezaei et al. 2022), which takes advantage of barcoding individually digested DNA samples, which are then pooled. One fraction of this pool is amplified to generate the GBS library used for the SNP analysis, while another fraction is subjected to Methylated DNA Immunoprecipitation and then amplified to generate the methylomic library. The detailed GBS‐MeDIP protocol has been previously published (Rezaei et al. 2022).

## 5 SNP (DNA) Analysis

### 5.1 Data Preparation and Filtering

The raw sequencing reads from GBS .fastq data for the SNP analysis were first subjected to quality control using FastQC (version 0.11.9) (Andrews 2010). Adapter removal and quality trimming were performed using Trimmomatic (version 0.39) (Bolger et al. 2014) with specified parameters for sliding window trimming, minimum length filtering, and Illumina adapter clipping. Paired‐end and single‐end reads were aligned to the reference genome (Galgal6) using BWA (version 0.7.17) (Li and Durbin 2009). The aligned reads were merged where applicable, and Picard (version 2.23.8) (https://github.com/broadinstitute/picard/) was used to add read groups, mark duplicate reads, and merge both paired and single‐end reads. Quality assessment was performed using Qualimap (version 2.2.1) (García‐Alcalde et al. 2012). Base quality score recalibration was carried out with GATK (version 3.8) (McKenna et al. 2010), followed by the creation of recalibrated BAM files. The final catalogue and variants were built using Stacks (ref_map.pl, version 2.41) (Catchen et al. 2013). The resulting variant call format (VCF) file contained SNP data from 34 samples across 36 chromosomes and was imported into R using the vcfR package (Knaus and Grünwald 2017). The dataset consisted of 3124 variants with no missing data. Population metadata was uploaded, and individuals were assigned to populations based on matching sample names using pop_apodemus data. The VCF data were converted into a genind object using the adegenet package (Jombart et al. 2018) to facilitate further genetic analyses. Genes with significant selection (Selection_Type == “Positive_Selection”) were filtered from the combined dataset, which also included genomic and annotation information. Gene sets were categorised based on different selection metrics (H‐L, H‐PO, L‐PO) associated with specific phenotypic contrasts. List columns were flattened by converting them to character strings to facilitate further analysis.

### 5.2 SNP Extraction and Annotation

All the sampled parental (*n* = 20), 5th generation high selection birds (*n* = 7), 5th generation intermediate birds (*n* = 4) and 5th generation low selection birds (*n* = 11) were then genotyped via the following protocol. The VariantAnnotation package was used to extract relevant SNP information, including reference (REF) and alternative (ALT) alleles. The genomic range (GRanges) of each SNP was also extracted. SNP data was filtered to include chromosomes (chr1 to chr28, chr30 to chr33, chrW, chrZ, and chrMT) using GenomicRanges (Carlson et al. 2014).

The reference genome (BSgenome.Ggallus.UCSC.galGal6) was used to sequence SNP regions, and CpG and CCpGG positions were identified. For CpG, the SNP region was extended by one base downstream, while for CCpGG, it was extended by one base upstream and two bases downstream. The sequences were analysed using the vcountPattern() function in Biostrings (Pages et al. 2013) to quantify CpG occurrences, and results were added to the SNP range data.

### 5.3 Functional Annotation

Annotations were performed using the ChIPseeker package (Yu et al. 2015) and the TxDb.Ggallus.UCSC.galGal6.refGene database. SNPs were annotated to gene features, including promoters, exons, and introns. Promoter regions were defined as 3 kb upstream and downstream of transcription start sites (TSS), and the annotatePeak() function was used to assign functional annotations to SNPs. The gene annotation database (org.Gg.eg.db) was used for functional assignment.

### 5.4 Principal Component Analysis (PCA)

A PCA was performed on allele frequency data generated by tabulating the genind object using dudi.pca() from the ade4 package (Chessel et al. 2004). Three principal components were retained for further analyses. The first three PCs results were visualised to assess genetic variation across samples.

### 5.5 Clustering Analysis

Clustering analysis was performed using find.clusters() from the adegenet package (Jombart 2008) to determine the optimal number of genetic clusters (K). Individuals were grouped into three genetic clusters, and a Principal Component Analysis was conducted to describe the genetic structure of the populations.

### 5.6 Fst and Genetic Differentiation

FST was calculated using Weir and Cockerham's estimator (hierfstat, Goudet 2005) on high‐quality SNPs (100% call rate) generated via GBS. SNPs in the top 5% (≥ 95th percentile) were classified as under positive selection, and those in the bottom 5% (≤ 5th percentile) as under balancing selection. A Manhattan plot was created using qqman to visualise Fst values across the genome.

### 5.7 Gene Enrichment Analysis

Enrichment analysis was conducted using the g:Profiler2 tool (version 0.2.3) (Kolberg et al. 2023) to identify overrepresented functional terms, including Gene Ontology (GO) categories, KEGG (Kyoto Encyclopedia of Genes and Genomes) pathways, Reactome pathways, and WikiPathways. The “ *Gallus gallus* ” organism database was used for annotation, and FDR correction was applied to control for multiple comparisons. Each gene set, as defined by selection metrics, was analysed separately. Only genes that were classified as being under significant positive selection were included in this analysis.

### 5.8 Visualisation

Cluster assignments from DAPC were visualised using compoplot from the adegenet package, which shows the membership probability of individuals in each genetic cluster. Barplots depicting sample sizes per population were generated to illustrate the distribution of samples. Results from the enrichment analysis were visualised using a customised dot plot created with the ggplot2 package in R (Wickham 2011). The plot displayed enriched functional terms for each gene set, with the size of the dots indicating the number of genes involved in each term and the colour representing the FDR‐adjusted *p*‐value. A red‐to‐blue gradient was used, with red indicating greater significance (lower FDR values).

## 6 DNA Methylation Analysis

### 6.1 Data Preparation and Filtering

The initial data preparation from GBS‐MeDIP .fastq data, including quality control, trimming, alignment, read group assignment, duplicate marking, and recalibration, was performed in the same manner as for SNP Analysis, using identical tools and parameters.

### 6.2 Peak Calling and Identification

Coverage peaks, referred to as regions of interest (ROIs), were identified using merged BAM files. The merged files were sorted and indexed using samtools (version 1.9). Peak calling for identifying these coverage peaks was performed using MACS2 (version 2.2.7.1) with broad peak identification and a FDR threshold of 0.1. Comparisons were conducted for various groups (e.g., HvsL, HvsP, IvsP, LvsP) to determine regions of significant coverage differences.

### 6.3 DMR Set Creation

Following peak identification, ROIs were used for DMR Set Creation. Regions were established using the MEDIPS.createROIset() function for different comparisons (e.g., H‐L, H‐P, I‐P, L‐P). The coupling factor for CpG sites was calculated using the MEDIPS.couplingVector() function, and differential methylation analysis was then conducted using MEDIPS.meth() for these ROIs.

### 6.4 MEDIPS Analysis

MEDIPS (v. 1.58.0) was used to further analyse the DMRs. Differential coverage analysis was performed using the MEDIPS.meth() function to identify significantly differentially methylated windows between experimental conditions (H‐L, H‐P, I‐P, L‐P). The edgeR method was applied for differential analysis, with FDR correction used to control for multiple testing. The functional annotation of DMRs was performed in exactly the same manner as described for SNP analysis.

### 6.5 Data Extraction and Post‐Processing

Significant regions (*p*‐value ≤ 0.05) were selected using MEDIPS.selectSig(). The selected significant DMRs were saved and exported for downstream analysis. Additionally, GRanges were created for all significant regions, and functional annotations were performed using ChIPseeker, similar to what was performed with the SNPs.

### 6.6 Visualisation and Analysis

Heatmaps were generated using the gplots and RColorBrewer packages to visualise differentially methylated regions between conditions. The heatmap.2() function was utilised with a red‐to‐blue colour gradient to indicate methylation changes.

## 7 Results

### 7.1 Intercross Population Analysis

Given this is an intercross population, the genotypes responsible for regulating brain composition, bodyweight, fear of humans, and anxiety in general should all be randomly distributed throughout the genome. This means that any phenotypic correlations should only occur if the loci for these different traits are either in linkage with one another or pleiotropic loci affect both traits simultaneously. All individuals share the same environment and rearing, so environmental considerations should be controlled for in this case. Note that this is an advantage over only comparing the selected populations. When comparing two selected or parental populations in isolation, any traits that have been inadvertently selected due to genetic drift, inbreeding, and the like will appear to be correlated, even if they have a distinct and non‐overlapping genetic architecture. In contrast, an intercross population will only show any correlations if the traits measured at least partly share a common or overlapping genetic architecture. The more generations that the intercross is interbred for, the more recombinations will build up, and the easier it will be to separate linkage from pleiotropy in cases where an overlap is seen. A total of 10,621 SNPs were generated for the QTL analysis.

### 7.2 Brain and Behavioural Correlations

To assess the relationship between brain size and brain composition with fear of humans behaviour, and fear behaviour in general, we performed a series of linear models assessing how brain size and brain composition predict fear of humans and fear behaviour (as measured by the flash test and the open field test). The only brain region to show any correlation with behaviour was the cerebellum. In the case of the cerebellum, there was a suggestive correlation with distance moved in the open field test (*p* = 0.06, *t*‐value = 1.9, *R* ^2^ = 0.03), and a suggestive correlation with latency to enter the central zone in the open field (*p* = 0.08, *t*‐value = −1.8, *R* ^2^ = 0.02). In addition, there was a significant correlation with AUC2 (the composite fear assessment test), with *p* = 0.04, *t*‐value = −2.1, *R* ^2^ = 0.03. All these results suggest that the cerebellum is somewhat correlated with fear behaviour, though there appears to be only a partial overlap in the genetic loci underlying these traits.

### 7.3 Brain and Bodyweight Correlations

Although brain size and the composition of brain sub‐regions are often correlated with body weight, as we have shown previously in an 8th generation wild × domestic advanced intercross (Henriksen et al. 2016), this correlation can often be eroded in such a population due to the potentially distinct genetic architectures between body weight and brain size and brain composition. Although we find that body weight is still correlated with brain composition and total brain size, the models with sex and body weight, body weight only, or sex only showed very similar *R*‐squared values, with the full model (sex and body weight) only adding between 2% and 8% increase in the *R*‐squared value, depending on the brain sub‐region under consideration (see Table 1). Further to this, we have previously shown that the genetic architectures for brain size and body size are non‐overlapping in at least one chicken intercross (Henriksen et al. 2016). Given this, only sex was used as a covariate for the brain size and composition QTL analysis, with body weight also assessed for QTL.

**TABLE 1:** *R*‐squared percentages for the three different linear models analysing the effects of sex and bodyweight on brain composition.

Brain region	Model type (%)		
	Sex only	Body weight only	Sex + bodyweight
Cerebellum	49	51	52
Cerebrum	40	48	48
Optic tectum	29	31	31
Midbrain	30	36	36
Total brain	47	55	55

### 7.4 Genetic Architecture

For fear of humans, a total of 14 QTL were identified, two of these being significant, with the remainder being suggestive (see Table S1). Multiple loci were also identified for brain size and brain composition, as well as proportional brain region size (cerebellum—2 significant, one suggestive, cerebrum—4 significant, 8 suggestive, optic tectum—2 significant, proportional cerebellum—2 significant, total brain size—6 significant, 6 suggestive). In addition, four suggestive loci for body weight were also found.

As well as the fear of humans assay, the two fear assays (open field and flash test) yielded a number of QTL. For the open field test, eight significant QTL and five suggestive QTL were identified (frequency to enter the centre—8 significant QTL were identified, latency to enter the centre—1 suggestive, latency to leave the start zone—3 suggestive, time in central zone—one suggestive). For the flash test, a large number of loci were identified (30 significant loci for AUC1, and two suggestive for the difference between AUC1 and AUC2).

### 7.5 Clustering of Brain and FOH QTL

What can immediately be seen with the detected genetic architectures is the large degree of overlap between loci for the different traits. In particular, QTL for fear of humans and brain size/brain region size strongly overlap into discrete clusters. Eight separate clusters display a close association/linkage with fear of humans and one or more brain composition QTL (see Table 2), whilst four clusters also showed linkage between anxiety‐related loci and brain composition loci.

**TABLE 2:** QTL clusters showing the co‐localisation of 21 separate clusters of QTL, plus the locations of QTL for brain weight derived from a separate wild × domestic intercross.

Study	Significance	Trait	Chrom	Position	Score	Effect	*R* ^2^	Cluster
High‐low	Suggestive	fear_score	1	106014811	3.95	1.4268781	0.019	1
High‐low	Significant	optic_tectum	1	110142757	3.97	0.0193883	0.134	1
High‐low	Suggestive	Cerebrum	1	113716393	2.65	0.04488	0.021	1
High‐low	Significant	total_brain	1	113716393	3.51	0.0883842	0.042	1
Wild‐domestic		Total brain weight	1	117882924–126047891				1
Wild‐domestic		Total cerebellum weight	1	125004874–131350907				1
High‐low	Suggestive	total_brain	1	170468749	2.79	−0.0872636	0.003	2
High‐low	Significant	AUC1	1	170468749	1.75	1.744459	0.009	2
High‐low	Suggestive	fear_score	1	177086581	3.39	1.0983948	0.01	3
High‐low	Suggestive	total_brain	1	177259328	2.73	−0.0836615	0.015	3
Wild‐domestic		Proportional cerebellum	1	177451389–181505126				3
High‐low	Suggestive	fear_score	1	193713501	3.75	1.3159411	0.013	4
High‐low	Suggestive	Cerebrum	1	195598686	2.72	−0.0458088	0.025	4
High‐low	Significant	total_brain	1	195598686	3.34	−0.0863197	0.027	4
Wild‐domestic		Total cerebellum weight	1	197455851–196261195				4
High‐low	Significant	AUC1	1	197585720	2.8	6.302186	0.048	4
High‐low	Significant	AUC1	3	3650776	2.52	−1.93916	0.029	5
High‐low	Significant	AUCdiff	3	3650802	4.39	3.08161	0.027	5
High‐low	Significant	Cerebrum	3	51266353	4.03	0.0894894	0.051	6
High‐low	Significant	total_brain	3	51266353	4.59	0.1608911	0.036	6
Wild‐domestic		Total cerebellum weight	3	55263726–62791236				6
Wild‐domestic		Total brain weight	3	57019681–62791236				6
High‐low	Significant	Cerebellum	4	10733319	3.94	0.046723	0.046	7
High‐low	Significant	total_brain	4	11200915	4.57	0.2350797	0.071	7
Wild‐domestic		Brooding	4	11346841–15229355				7
High‐low	Significant	Cerebrum	4	11440257	4.96	0.1677873	0.071	7
High‐low	Suggestive	fear_score	4	12333824	4.1	1.0757852	0.003	7
High‐low	Significant	AUC1	5	9838327	2.21	−6.734805	0.114	8
Wild‐domestic		Proportional cerebellum	5	13243005–19572670				8
High‐low	Significant	freq_centre	5	36048089	2.95	4.446599	0.035	9
High‐low	Significant	AUC1	5	36048089	2	−3.752325	0.007	9
High‐low	Significant	freq_centre	7	10695203	3.06	2.321429	0.059	10
Wild‐domestic		Proportional cerebellum	7	15205783–18209201				10
Wild‐domestic		Total cerebellum weight	7	15205783–22616032				10
High‐low	Suggestive	Cerebellum	7	34773780	3.04	0.0246726	0.002	11
High‐low	Suggestive	Cerebrum	7	35732524	2.85	0.1144189	0	11
High‐low	Significant	total_brain	7	35732524	2.94	0.1958357	0.001	11
High‐low	Significant	fear_score	7	36163163	4.89	0.8000769	0.05	11
High‐low	Suggestive	fear_score	9	3496532	3.4	1.022221	0.008	12
Wild‐domestic		Total brain weight	9	3692046–5986625				12
High‐low	Significant	Relcerebellum	9	4974949	2.72	−0.0058309	0.055	12
Wild‐domestic		Total cerebral hemisphere weight	10	16035116–19056293				13
High‐low	Significant	Cerebrum	10	20347202	4.42	0.0816204	0.08	13
High‐low	Significant	total_brain	10	20347202	3.53	0.1215934	0.025	13
High‐low	Suggestive	Cerebrum	13	1613639	3	0.050188	0.052	14
High‐low	Significant	total_brain	13	1613639	2.91	0.0817117	0.025	14
Wild‐domestic		Brooding	13	3424796–6538686				14
High‐low	Significant	freq_centre	13	9292610	3.26	3.708639	0.09	15
High‐low	Significant	AUC1	13	9372064	1.85	−2.328819	0.03	15
High‐low	Significant	optic_tectum	13	16488098	3.44	0.0505432	0.167	16
High‐low	Significant	Cerebrum	13	16750632	4.98	0.1608468	0.025	16
High‐low	Suggestive	total_brain	13	16750632	4.83	0.2669036	0.024	16
High‐low	Significant	Cerebellum	13	17148764	4.81	0.0483338	0.043	16
High‐low	Significant	freq_centre	15	6549886	2.87	−4.627744	0.075	17
High‐low	Suggestive	lat_start	15	6888610	4.44	85.03995	0.119	17
High‐low	Suggestive	lat_centre	15	6888610	4.77	87.596	0.207	17
High‐low	Suggestive	AUCdiff	20	10347956	3.13	3.857699	0.023	18
High‐low	Significant	AUC1	20	12486120	1.66	1.678272	0	18
High‐low	Suggestive	fear_score	23	2571424	3.75	1.1487806	0.072	19
High‐low	Suggestive	Cerebrum	23	2959723	2.89	0.0899678	0.015	19
High‐low	Significant	Relcerebellum	23	4277592	2.87	0.003288	0.087	19
High‐low	Significant	AUC1	23	5529285	2.33	1.47744	0.038	19
High‐low	Significant	AUC1	24	1448228	2.92	2.810711	0.006	20
High‐low	Suggestive	Cerebrum	24	4690366	2.72	0.2178478	0	20
High‐low	Suggestive	fear_score	24	5359235	3.47	1.1110066	0.001	20
High‐low	Significant	freq_centre	27	4082079	3.15	6.346157	0.016	21
High‐low	Significant	AUC1	27	7775021	2.14	2.66103	0.002	21

*Note:* Chromosome (chromosome QTL is located on), position (position in bp), QTL effect (additive effect of the QTL), QTL effect size (*R*‐squared % explained by the QTL), and significance score (significance score for each QTL) are all given. In addition, the study is also given, with High‐Low indicating the intercross results for this study, and wild‐domestic referring to the results obtained from the wild × domestic advanced intercross published previously.

### 7.6 Repeatable Brain Loci With Domesticated Birds

In addition to the clusters for fear of humans and brain composition, it was also possible to overlay these results with those from a previous study looking at the genetic loci for brain composition and brain size in a wild × domestic intercross. In this case, we once again see a very strong overlap between the loci for brain composition between the two studies, with five clusters containing adjacent or overlapping brain composition loci from the two studies, as well as fear QTL (clusters 1,3, 4,6,12), and with two clusters showing an overlap between only fear behaviour (the open field test and the startle test) and total and proportional cerebellum size (clusters 8 and 10), see Table 2. In total, 13 of the 20 loci for brain and brain composition from the previous studies overlapped with the clusters detected here.

### 7.7 Differentially Epigenetically Marked Genes Present in the Selected Lines Overlap With Fear of Human and Brain Composition QTL

A total of five of the 25 genes that were found to be differentially methylated between high and low fear selected parental birds, or differentially methylated between the selected birds and the unselected P_0_ generation, were found to overlap with the QTL identified for fear of humans, brain composition, or anxiety behaviour in the selected intercross (see Table 3 and also the following section). Of these, two overlapped with two of the clusters combining fear of humans and brain composition (*NROB1, MIR1600*).

**TABLE 3:** Genes present in high‐relevance differentially methylated regions that are present in QTL regions.

Comparisson	DMR location	geneChr	geneStart	geneEnd	geneLength	geneStrand	geneId	transcriptId	distanceToTSS	ENSEMBL	GENE	CLUSTER
S5 High vs. P0	chr1:115590864–115591029	1	115748153	115750190	2038	2	395285	NM_204593	159,161	ENSGALG00000016287	NR0B1	1
S5 Low vs. P0	chr1:197591338–197591628	1	197485636	197485715	80	2	100315959	NR_035086	−105,623	ENSGALG00000025438	MIR1600	4
S5 Low vs. P0	chr8:7420609–7420743	8	7387437	7436311	48875	1	396213	NM_205276	33,172	ENSGALG00000004526	TNR	—
S5 Low vs. P0	chr11:18562255–18562373	11	18523346	18544326	20981	2	415846	NM_001030580	−17,929	ENSGALG00000032410	CBFA2T3	—
S5 High vs. S5 Low	chr11:18686930–18687047	11	18682695	18682796	102	2	100315996	NR_035286	−4134	ENSGALG00000036130	MIR1785	—

*Note:* Specific comparison where the DMRs are located, gene start and end, ENSEMBL gene name and abbreviation are given.

## 8 Genetic and Epigenetic Differences Between the Sperm DNA of the Selected and Parental Lines

We investigated the sperm genome and methylome of parental (P0) and fifth generation selected (S5H, S5I, S5L) individuals to shed light on the effects that selection can have on the male germ line, considering its critical role in inheritance.

### 8.1 Genetic Differences

**FIGURE 1:**
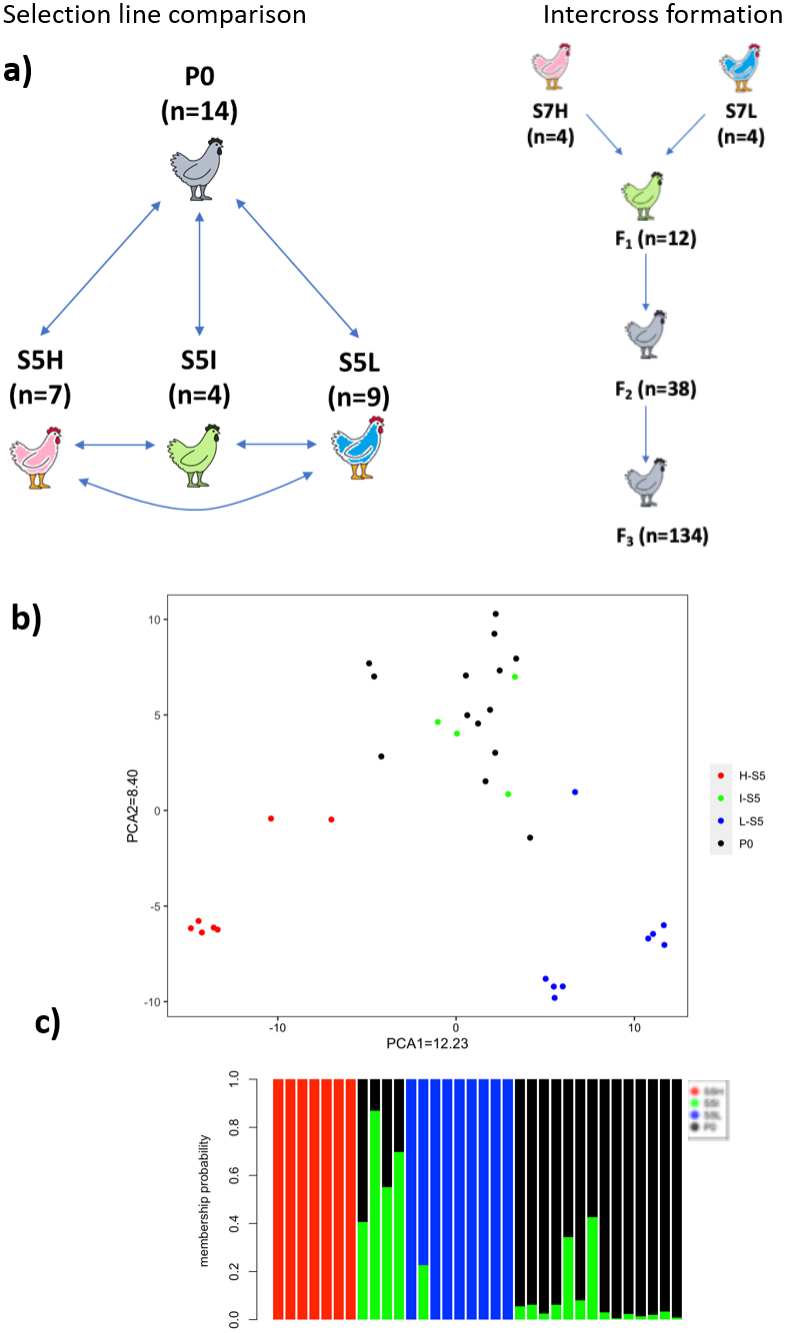
(a) Overview of the samples used for differential Selection Line Comparison analysis and the formation of the intercross. S5H, S5I, S5L denote the fifth generation High, Intermediate and Low selection lines respectively, whilst S7H and S7L denote the seventh generation high and low selection lines, respectively. (b) PCA clustering of the selection lines and the starting (P0) population. The % variance explained by each PC is given after its name in the legend. (c) Cluster analysis of the selection lines and starting population, showing four independent groups.

**FIGURE 2:**
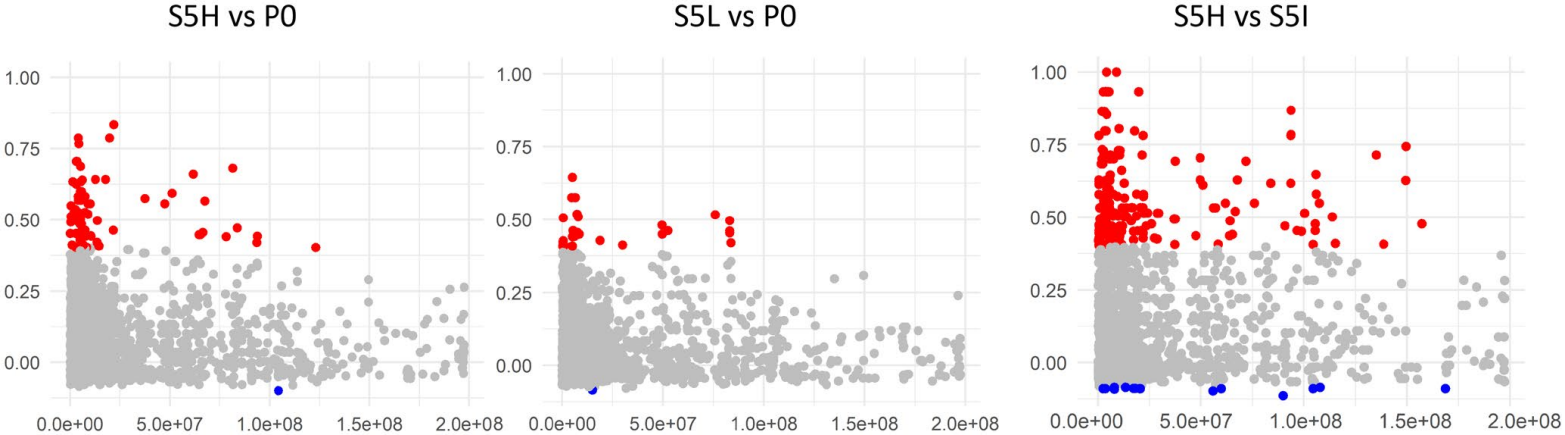
SNPs under balancing, neutral, and positive selection in population comparisons between the selection lines (high selection line = S5H, low selection line = S5L) and the starting generation (P0). *Y*‐axis represents the Fst score.

We filtered the SNPs using a 100% allele and individual call rate. After this filtering, we obtained 3124 SNPs among the 34 individuals, 14 P0, 7 S5H, 4 S5I, and 9 S5L (Figure 1a). Discriminant analysis of principal components (DAPC) using these SNPs resulted in successful categorisation of the individuals within their respective groups, showing a large separation of the individuals in the selected lines (S5H and S5L) compared to the P0 and S5I individuals, which are slightly separated (see Figure 1b). Membership probability was also calculated with these 3124 SNPs, which resulted in high membership probability for the selected lines (S5H and S5L), while S5I individuals showed a medium probability of also belonging to the P0 group (Figure 1c). We performed FST analyses using the 3124 SNPs obtained with 100% call rates to distinguish between different types of selective pressures acting on our experimental groups, specifically whether marks of balancing or positive selection, or neutral evolution could be identified. Although the populations were bred for only five generations, the extended linkage disequilibrium in chickens supports the detection of selection signatures with this approach. We acknowledge that genetic drift may influence FST values; however, our stringent filtering minimises this effect. One additional caveat that should be noted here is that as GBS is a reduced representation technique (i.e., only a subset of the genome is used), the number of SNPs used in this analysis is somewhat limited. This may result in the failure to identify gene regions under positive or negative selection due to insufficient coverage. Despite this, we do manage to identify some gene regions of positive and negative selection. We restricted the FST analysis to the comparisons between the selection lines and the P0 generation, and the selection lines and the S5I generation. FST analysis between the different experimental groups displays the strong effect of positive selection on the selected lines (S5H and S5L) compared to P0 (Figure 2, and Table S2). In the case of the S5H‐P0 comparison, 101 SNPs were under positive selection with only 1 SNP under balancing selection. For the S5L‐P0 comparison, 36 SNPs were under positive selection, and once again with only a single SNP under balancing selection. In the comparison between the S5H and S5L populations, we found 18 SNPs under balancing selection, and 276 SNPs under positive selection. We also assessed the number of genes present in these positively selected regions: in the S5H‐P0 comparison, 69 genes were associated with positively selected SNP regions, whilst in the S5L‐P0 comparison, 29 genes were present in positively selected SNP regions. Using a GO analysis performed on the genes in these positively selected regions, we find an enrichment of terms principally involving immune‐related and apoptosis in the high selection line, and immune and growth/cell structure in the low selection line (see Table 4). In addition, we also checked for an overlap between the genes present in the 98 SNPs under positive selection and the QTL identified in the intercross. Using a window of ±2 Mb from the significant QTL SNP, we found that 20 of the genes were present in the QTL regions. These genes were *POLR2C, GFOD2, CHMP1A, MIR1636, HIP1R, CRYBB2, CRYBA4, ASS1, EXOSC2, EPB41, MEAF6, CSF3R, ADPRHL2, COL9A2*.

**TABLE 4:** Gene ontology analysis.

Comparison	*p*	term_size	query_size	term_id	term_name
S5H_PO	0.032	2	50	GO:0140367	Antibacterial innate immune response
S5H_PO	0.047	10,232	59	TF:M00646	Factor: LF‐A1; motif: GGGSTCWR
S5H_PO	0.047	1813	59	TF:M07054	Factor: P53; motif: NNNNNACAWGCCYNNN
S5L_PO	0.046	91	20	GO:0019838	Growth factor binding
S5L_PO	0.046	1	20	GO:0035757	Chemokine (C‐C motif) ligand 19 binding
S5L_PO	0.046	1	20	GO:0035758	Chemokine (C‐C motif) ligand 21 binding
S5L_PO	0.046	1	20	GO:0038117	C‐C motif chemokine 19 receptor activity
S5L_PO	0.046	1	20	GO:0038121	C‐C motif chemokine 21 receptor activity
S5L_PO	0.01	23	11	KEGG:03020	RNA polymerase
S5L_PO	0.01	200	11	KEGG:04810	Regulation of actin cytoskeleton
S5L_PO	0.015	114	4	WP:WP824	Regulation of actin cytoskeleton

*Note:* Table showing significant GO (Gene Ontology), KEGG (Kyoto Encyclopedia of Genes and Genomes), WP (WikiPathways) and TFs (Transcription Factor) terms derived from the positively selected genes identified in the selection line comparisons performed between P0 and S5 generation animals. Significance (5% FDR‐adjusted for multiple testing), and the terms derived are presented. The comparison column indicates in which of the three comparisons the terms were significant, and the size of the terms are also given in addition.

### 8.2 Methylomic Differences

For the methylomic comparisons we used two levels of stringency in our analysis. One less stringent analysis identified Differentially Methylated Regions (DMRs) based on differential regions of interest (ROI) identified by MACS2 (Adj *p* ≤ 0.05); these were named Differentially Methylated Regions of Interest (DMROIs). This analysis was done to identify general patterns among the experimental groups. Additionally, a more stringent analysis involved performing a *t*‐test (*p* ≤ 0.05) among experimental groups based on the previously identified DMROIs. This was done to pinpoint specific DMRs of high relevance (hrDMRs).

Our DMROI analysis (Figure S1 and Table S3) revealed 133 regions between P0 and S5H (100 hypermethylated in S5H), and 77 regions between P0 and S5L (54 hypermethylated in S5L). This shows a strong tendency for sperm hypermethylation as selection progresses from P0 to S5, independent of the selection line. We also observe a higher total number of regions in the two groups subjected to positive selection (S5H and S5L) compared to S5I, subjected to balancing selection. Additionally, we found 114 regions between S5H and S5L, with many more hypermethylated regions in S5H (99), showing that sperm hypermethylation mostly relates to selection for high fear of humans.

Our hrDMRs (Figure S2c) revealed 11 regions between P0 and S5H (all hypermethylated in S5H), and 14 regions between P0 and S5L (13 hypermethylated in S5L). Thus, we again find a strong tendency for sperm hypermethylation as selection progresses from P0 to S5, independent of the selection line. Additionally, we found 10 regions between S5H and S5L, all hypermethylated in S5H, showing that even in the DMRs obtained among the extreme groups, sperm hypermethylation mostly relates to selection for high fear of humans. The individual variation in DNA methylation levels can be observed in the heat maps of Figure S2.

## 9 Discussion

In this study we find that brain composition, fear of humans, and anxiety behaviour show a high degree of variability in the intercross population, as expected given the divergent selection present in the parental generations used. We find that the genetic loci fear of humans, brain composition, and anxiety all closely overlap in a series of discrete clusters. Further, these loci also overlap with the loci detected for brain composition in a previous wild × domestic intercross (Henriksen et al. 2016). We also assessed the differences between the selection lines themselves, via the analysis of genes that were under positive selection and partially or fully fixed. Positively selected SNPs obtained in sperm between the P0 and S5 individuals were found in 98 genes. The genes present in these regions were primarily related to the immune system and apoptosis (high selection line), or growth and the immune system (the low selection line). Sperm DNA methylation differences between the same individuals identified 25 Genes that appeared to be differentially methylated between the selection lines, which mostly exhibit increases in methylation with selection, particularly in the high fear line.

Perhaps the most striking results from this study are these clustered effects of fear of humans, anxiety, and brain composition. Although the resolution of this intercross is insufficient to disentangle pleiotropy from close linkage, it is notable that such a co‐localisation occurs. This indicates that the responses to selection on fear of humans derive from changes in brain composition, or at the very least that the loci regulating fear of humans are linked to those loci responsible for regulating brain composition. If these loci are genuinely pleiotropic, it would indicate that these regions (the cerebellum in particular, but also the cerebrum) potentially regulate fear of humans behaviour. We have previously shown that the cerebellum is potentially involved in regulating brooding behaviour (Henriksen et al. 2016). In addition to this, we once again demonstrate that different brain regions (the cerebrum, cerebellum, etc.) have different loci regulating them, thus providing further evidence for the mosaic theory of brain evolution and development, whereby different brain regions can be regulated independently of one another.

Our findings also have a bearing on the domestication syndrome and its ontogeny, occurrence and persistence. However, it has been observed that many of the traits linked to the domestication syndrome are not common to all (Wright et al. 2020a; Johnsson et al. 2021). Further to this, some authors have also suggested that the domestication syndrome does not actually exist due to the low number of holistic changes common to all domestic animals and that these traits did not arise all at the same time (i.e., they should be pleiotropic to be considered part of the domestication syndrome) (Lord et al. 2020a, 2020b). However, Wright et al. (2020a) identified that actually the most consistent domestication syndrome traits were behavioural changes (tameness/reduced fear of humans) and brain composition alterations. In this case, pleiotropy did not necessarily have to occur for traits to be considered part of the domestication syndrome; however, linkage would facilitate in ensuring these traits were easily passed on together. In this case of the experiment presented here, our results appear to indicate that the two most basal traits for the domestication syndrome could well arise simultaneously, either due to pleiotropy or close linkage. This would suggest that the remaining domestication traits would slowly accrue afterwards, but that solely selecting on tameness can give rise to at least two of the domestication syndrome traits (Agnvall et al. 2015).

The QTL clusters detected in this study appear to mirror the clusters that arise during domestication in the chicken, with a potentially pleiotropic core surrounded by linked loci of the different traits associated with domestication. Evidence for this comes from studies on a domesticated × wild chicken intercross (Johnsson et al. 2012, 2018a, 2018b, 2015a, 2014; Wright et al. 2008, 2006, 2012, 2010). For example, clusters containing QTL for comb size, fecundity, bone allocation, and egg production (Johnsson et al. 2012, 2014, 2015b; Wright et al. 2006) loci were found to co‐localise in discrete clusters, with several other examples for other traits also identified (Johnsson et al. 2018a, 2016a). Therefore, the early selection for tameness already recapitulates the clustered loci seen in much older domesticated lineages and would suggest these form early on during the domestication process (although are no doubt supplemented with additional loci as domestication progresses).

These clusters have been proposed as the mechanism for the progression of domestication (Wright 2015; Grant 1981), in contrast to the model that suggests pleiotropy is the main driver of domestication‐related traits (Belyaev 1979; Belyaev et al. 1985), or gene networks (Trut et al. 2009). The problem with pleiotropy as the main driver of domestication is that when advanced intercrosses (that reduce linkage due to greater numbers of recombinations and hence increase the resolution of detected QTL) and gene expression analysis of expression QTL were performed in the chicken, these indicate that pleiotropy is less common than simply linkage between traits and pleiotropic eQTL ‘hotspots’ (where a specific locus regulates many different genes) are not common (Johnsson et al. 2015b, 2016a; Heyne et al. 2014; Höglund et al. 2020b). In the case of clusters, these have previously been thought to arise in domestication due to the effects of selection and then repeated introgression of new characteristics or traits (Grant 1981). In this model, those loci in linkage with one another are easier to recapitulate and fix once again after the introgression of certain favourable traits and hence are why it is very easy to rapidly recapitulate the domesticated phenotype after such an introgression.

The study presented here, with clusters arising after only eight generations of selection, would indicate that these loci seem to already segregate together, and that the loci for these traits are not randomly distributed throughout the genome. Further, if these loci are genuinely pleiotropic, they could give some evidence for the neural crest theory of domestication (Johnsson et al. 2021; Wilkins et al. 2014). This theory posits that domestication traits are derived from neural crest cells, and changes in neural crest cell proliferation and migration are causal to the variety of different domestication characteristics. Evidence for this hypothesis is somewhat equivocal (Johnsson et al. 2021), the study presented here could indicate that the core of the domestication traits could be derived from neural crest cells if they are pleiotropic, though with no evidence either way for the more outlying loci in the clusters.

Remarkably, the loci selected during artificial selection for tameness appear to select for the same (or rather potentially the same genes or genetic loci) that were selected during actual domestication, as evidenced by the close overlap between the QTL for brain composition identified in a wild × domestic intercross and those in this selected intercross. As yet, it is impossible to ascertain if the same polymorphisms (which seems unlikely) or even if the same genes are involved. Only by resolving the precise genes and/or polymorphisms involved in both intercrosses can this be confirmed. However, it does appear that if not the same genes, then very similar regions are regulating these traits in the two intercrosses, and this would imply that domestication selection is at least partly repeatable. In many ways, this repeatability mirrors the repeatability that we see in the opposing selection pressure of feralisation (where domestic animals escape or are released into the wild) (Gering et al. 2019a, 2019b; Henriksen et al. 2018). In the case of feralisation in the chicken, it has been shown that feralisation selection acts through different genes/polymorphisms than domestication, and is not simply a process of domestication in reverse (Martin Cerezo et al. 2023; Johnsson et al. 2016b). However, feralisation itself in the chicken does appear to be at least partly repeatable, with chickens from Bermuda and Hawaii, despite sharing no gene flow, showing significant overlap of signatures of selection (selective sweeps) (Gering et al. 2024). This would be another example of how the targets of rapid selection are repeatable, even with different starting populations.

The analysis of the intercross population has allowed us to assess the degree of overlap between loci and also between crosses; however, the limited resolution of an *F*_2_/*F*_3_ intercross means that identifying genes or pathways is limited. In this case, however, we have the genetic and epigenetic analyses of sperm in the founder populations compared to the selected lines to allow some inferences to be performed. The sperm genomic and methylomic analyses allow us to be more certain that these differences are due to the selection process and are heritable. In the epigenetic analyses, we find that five of the 25 significantly differentially methylated genes co‐occur in QTL regions (NROB1, TNR, MIR1600, CBFA2T3, MIR1785) and are therefore potentially causal genes to the selection for tameness. These genes have somewhat diverse functions, but involve adrenal (Achermann and Vilain 1993; Fan et al. 2021) and reproductive development (Suntharalingham et al. 2015), neural development (Wagner et al. 2020), lesions in the CNS (Roll and Faissner 2019), bone formation (Yao et al. 2019), stress regulation (Kim et al. 2024), and cognitive decline (Dou et al. 2019).

Genetic differences between the lines also help to identify the types of genes and pathways that are under selection. SNPs in 98 genes were found to be under positive selection in the sperm, between P0 and S5 individuals. Among them, one gene of special interest is TLR4, which has been previously reported to associate with positive selection in birds. TLR4 is involved in immune response (Grueber et al. 2014; Shultz and Sackton 2019), with its expression correlating to resistance to *Salmonella* infection in chickens, mice, and humans (Chaussé et al. 2011). Concordantly, GO analysis found enrichment for immune‐related genes and apoptosis in the high selection line, and immune and growth/cell structure in the low selection line. This shows some degree of overlap with the GO terms identified by looking at gene expression differences between these birds in hypothalamus tissue. In this case, cellular components and mitochondrial processes were most enriched, but when considering the 21 genes that were found to be significantly differentially expressed between the lines, immunological and reproduction‐related functions were the main traits involved (Bélteky et al. 2016). In this regard, the results appear to tally between the gene expression results in the hypothalamus as compared to the differential methylation analysis on sperm from the same generations.

One issue that can be problematic when comparing domestic populations with their wild counterparts, as a proxy to their wild progenitors, comes from just how good a proxy these modern wild populations are to the original progenitors. At the very least, these modern wild populations have undergone evolution in tandem with the domestic progenitor. They can also be introgressed to a greater or lesser degree with the domestic line due to interactions with feral populations (Wright et al. 2020a; Lord et al. 2020a, 2020b). The Red Junglefowl used for the selection experiments (and for the intercross) were obtained from European zoos, and therefore likely from populations somewhat habituated to humans before being moved to our lab. We know from previous studies (Håkansson et al. 2007) that some aspects of behaviour such as tameness and temperament may differ somewhat between different zoo populations. However, we have also shown that these populations are nevertheless quite representative of the ancestral, wild Red Junglefowl that they are highly useful as a basis for comparisons with present‐day domesticated birds (Schütz et al. 2001). Furthermore, in order to maximise genetic variation in the parental generation of the selected lines of Red Junglefowl, the birds were outcrossed between two different zoo populations before the onset of the selection (Agnvall et al. 2012).

In summary, our study demonstrates the importance of selection for tameness in driving early domestication traits, and indicates that the changes in brain composition can be linked with tameness behaviour in the chicken. Furthermore, some of the DNA methylation changes observed in sperm in the 5th generation of selection are present in QTL detected in the 8th generation that were subsequently intercrossed. Although we only examine the chicken in this study, taken together these results could help to explain why the most consistent traits in the domestication syndrome in almost all domestic animals are behavioural and brain compositional changes.

## Author Contributions

P.J. formulated the study. R.K., R.H., and D.W. collected the phenotypic data. D.W., F.P., D.W., and Z.M. generated the genetic and epigenetic data. C.G.‐B., F.P., M.L.M.C., R.H., and D.W. performed the analysis. P.J., D.W., and C.G.‐B. wrote the initial draft of the manuscript, all authors then edited and corrected this.

## Conflicts of Interest

The authors declare no conflicts of interest.

## Supporting information


**Figure S1.** (a) Differential methylation analysis of the different selection lines and P0 generation. (b) Number of hypermethylated regions as a proportion of total differentially methylated regions in the DMR analysis. (c) Number of hypermethylated regions as a proportion of total differentially methylated regions in the high relevance DMR analysis. The dotted line indicates the significance threshold.

**Figure S2.** (a–d) Heat maps of the differentially methylated regions, showing specific individuals methylation levels for each comparison. Colours equate to the degree of DNA methylation, with bluer colours indicating increased methylation and whiter (lighter) colours indicating less methylation.

**Table S1.** QTL detected via GWAS analysis. Significance (significant or suggestive), trait, type of model, threshold for significance, chromosome, position, SNP score (*p*‐value), *R*‐squared of the SNP, and model *p*‐value (*p*‐value of each significant SNP when combined with all other significant SNPS) are given.

**Table S2.** All SNPs tested for Fst and selection and their scores in each of the different comparisons. Chromosome, bp position, Fst value, type of selection, and annotation and metrics of any gene present in the region are all given.

**Table S3.** Significant high relevance Differentially Methylated Regions, the comparison in which they were significant, their location, *p*‐value, and gene metrics for any genes located within the region, are all given.

## Data Availability

The QTL mapping data for the intercross birds is available at http://doi.org/10.5061/dryad.fttdz094r on Dryad, whilst the SNP and DNA methylation information for the inter‐line comparisons is available at https://www.ebi.ac.uk/ena/data/view/PRJEB89427 on the ENA repository.
