# Supplementary figures and images for: Selection for Tameness in Red Junglefowl Recapitulates Genetic Loci Associated With Domestication‐Related Brain Composition
Source: Mol Ecol. 2025 May 19;34(24):e17788. doi: 10.1111/mec.17788 (PMC12717969; doi:10.1111/mec.17788)

a)

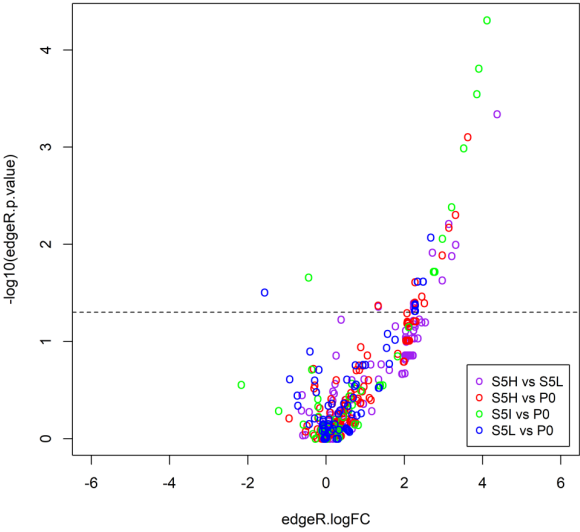

b)

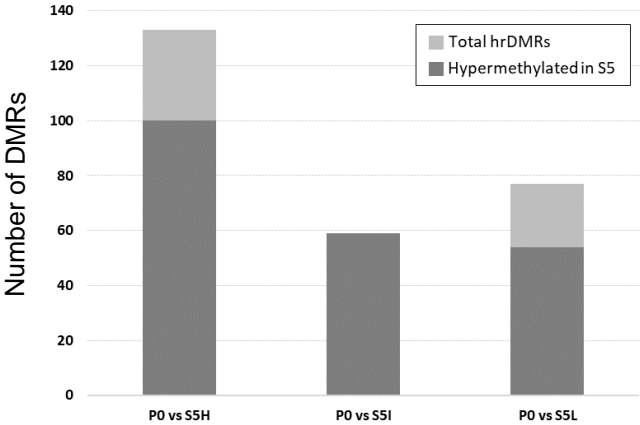

c)

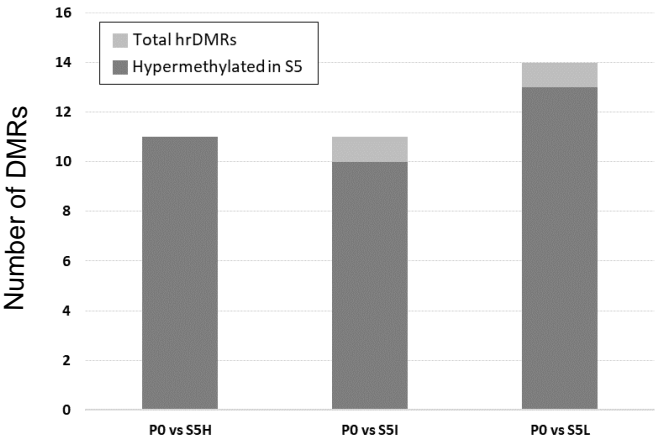

Supplement: Supplementary file 1 — Figure S1. (a) Differential methylation analysis of the different selection lines and P0 generation. (b) Number of hypermethylated regions as a proportion of total differentially methylated regions in the DMR analysis. (c) Number of hypermethylated regions as a proportion of total differentially methylated regions in the high relevance DMR analysis. The dotted line indicates the significance threshold. [file MEC-34-e17788-s004.pdf]

a) S5H vs P0

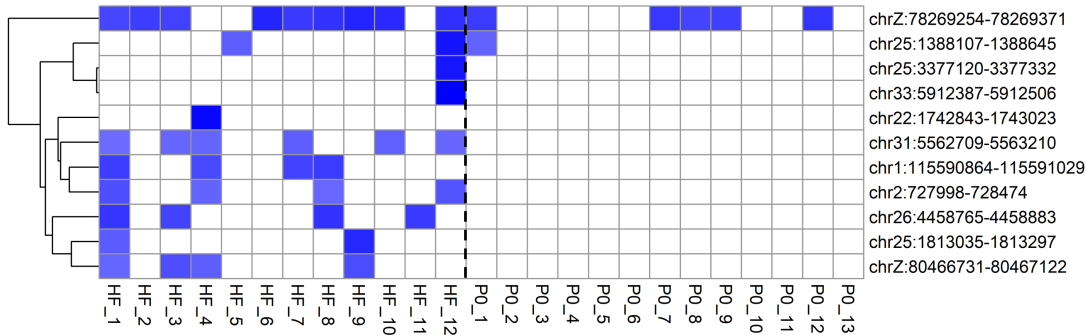

b) S5I vs P0

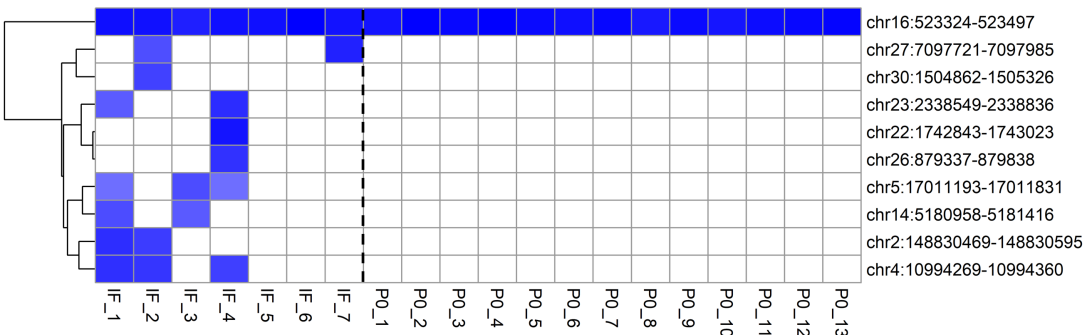

c) S5L vs P0

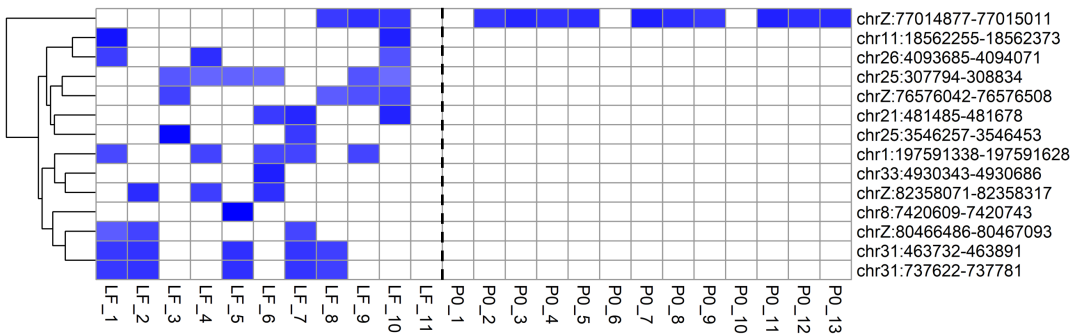

d) S5H vs S5L

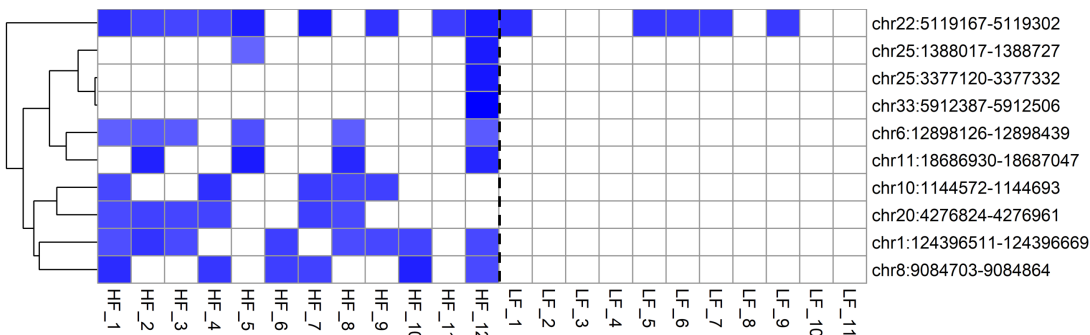

Supplement: Supplementary file 2 — Figure S2. (a–d) Heat maps of the differentially methylated regions, showing specific individuals methylation levels for each comparison. Colours equate to the degree of DNA methylation, with bluer colours indicating increased methylation and whiter (lighter) colours indicating less methylation. [file MEC-34-e17788-s005.pdf]
